# Harnessing chickpea bacterial endophytes for improved plant health and fitness

**DOI:** 10.3934/microbiol.2024024

**Published:** 2024-07-08

**Authors:** Yulduzkhon Abdullaeva, Gulsanam Mardonova, Farkhod Eshboev, Massimiliano Cardinale, Dilfuza Egamberdieva

**Affiliations:** 1 Institute of Molecular Microbiology and Biotechnology, University of Münster, Münster, Germany; 2 Faculty of Biology, National University of Uzbekistan, Tashkent 100174, Uzbekistan; 3 S. Yu. Yunusov Institute of the Chemistry of Plant Substances, Academy of Sciences of Uzbekistan, Tashkent 100170, Uzbekistan; 4 Department of Biological and Environmental Sciences and Technologies–DiSTeBA, University of Salento, Lecce, Italy; 5 Institute of Fundamental and Applied Research, National University of Uzbekistan TIIAME, Tashkent 100000, Uzbekistan

**Keywords:** chickpea, biotic stresses, drought, salinity, pathogens, plant traits

## Abstract

Endophytic bacteria live asymptomatically inside the tissues of host plants without inflicting any damage. Endophytes can confer several beneficial traits to plants, which can contribute to their growth, development, and overall health. They have been found to stimulate plant growth by enhancing nutrient uptake and availability. They can produce plant growth-promoting substances such as auxins, cytokinins, and gibberellins, which regulate various aspects of plant growth and development. Endophytes can also improve root system architecture, leading to increased nutrient and water absorption. Some endophytes possess the ability to solubilize nutrients, such as phosphorus and potassium, making them more available for plant uptake, and fixing atmospheric nitrogen. Chickpea (*Cicer arietinum*) is a major legume crop that has mutualistic interactions with endophytes. These endophytes can benefit the chickpea plant in various ways, including higher growth, improved nutrient uptake, increased tolerance to abiotic and biotic stressors, and disease suppression. They can produce enzymes and metabolites that scavenge harmful reactive oxygen species, thus reducing oxidative stress. Moreover, several studies reported that endophytes produce antimicrobial compounds, lytic enzymes, and volatile organic compounds that inhibit the growth of fungal pathogens and trigger systemic defense responses in plants, leading to increased resistance against a broad range of pathogens. They can activate plant defense pathways, including the production of defense-related enzymes, phytoalexins, and pathogenesis-related proteins, thereby providing long-lasting protection. It is important to note that the diversity and function of chickpea-associated endophytes can vary depending on factors such as variety, geographical location, and environmental conditions. The mechanisms behind the plant-beneficial interactions are still being intensively explored. In this review, new biotechnologies in agricultural production and ecosystem stability were presented. Thus, harnessing chickpea endophytes could be exploited in developing drought-resistant cultivars that can maintain productivity in arid and semi-arid environments, crucial for meeting the global demand for chickpeas.

## 1. Introduction

The importance of microorganisms in plant health and survival is well-recognized. Plant root can establish interactions with its associated microbes as so-called endophytes, which include rhizobia, arbuscular mycorrhizal fungi (AMF), and plant growth-promoting rhizobacteria (PGPR); these microbes can benefit their host plants in several ways, including plant growth stimulation, improved nutrient acquisition, regulating plant hormone levels, and improved disease resistance against pathogens [1]–[3]. Although the interactions between plants and endophytes have been extensively investigated, there is still limited knowledge of the mechanisms used by plant endophytic microorganisms to mitigate various abiotic and biotic stresses [4].

Chickpea (*Cicer arietinum*), with a total production of about 18.09 million tons (FAO STAT, 2022), is the third-most-produced legume grain after the common bean and soybean. Along with the production of different bioactive compounds, it is recognized as rich in protein, fat, minerals, and vitamins [5]. Chickpea is particularly important in arid lands, where productivity is severely restricted by salt stress and drought [6]. Chickpea can withstand such adverse conditions with the support of its microbiome, which includes genera like *Bacillus*, *Pseudomonas*, *Azorhizobium*, *Bradyrhizobium*, *Ensifer*, *Mesorhizobium*, and *Rhizobium*, allowing for the production of high yields in numerous nitrogen-poor and hostile soils [7]–[9]. It can also establish beneficial symbiotic relationships with AMF, which are known to effectively reduce drought stress by a variety of mechanisms including the control of plant hormonal balance, an increase in photosynthesis rates and leaf gas exchange, and the facilitation of water movement from the soil to the plant via extraradical mycelium [10]. However, environmental factors including drought, salt stress, and extreme changes in temperature can hinder the establishment of these relationships [11]. For example, drought has a significant detrimental effect on nodule functioning because it causes early maturity and a decrease in N fixation [12],[13]. Furthermore, salt stress restricts plant development by shrinking nutrient uptake, thus making plants more vulnerable to soil-borne diseases [14],[15]. In such conditions, plant-associated endophytes protect plants from oxidative stress, stimulate plant growth, improve physiological processes, and protect them from various soil-borne pathogens [16]–[19].

The traits involved in plant-growth stimulation and tolerance to abiotic and biotic stresses by endophytic bacteria have been commonly reported and reviewed in previous studies [20]–[24]. The majority of bacteria are able to produce various biological active compounds including phytohormones such as auxin, gibberellins, jasmonate, cytokinin, Indole-3-acetic acid (IAA) [25], siderophores [26], 1-aminocyclopropane-1-carboxylate (ACC) deaminase [27], as well as antifungal or antibacterial agents [28].

**Table 1.** Bacterial and fungal endophytes were identified in different tissues of the chickpea (*Cicer arietinum*).

Bacterial and fungal endophytes	Plant tissue	Reference
*Mesorhizobium*, *Burkholderia*, *Bacillus*, *Priestia*, *Paenibacillus*, *Alcaligenes*, *Acinetobacter*, *Rahnella*, *Enterobacter*, *Microbacterium*	seed	[29]
*Enterobacter* sp., *Bacillus* sp., *Pseudomonas* sp., *Staphylococcus* sp., *Pantoea* sp.	seed	[25]
*Bacillus*, *Sphingomonas*	seed	[30]
*Rhizobium leguminosarum* subsp. *ciceri*	root nodules	[31]
*Mesorhizobium* sp.	root nodules	[32]
*Mesorhizobium*, *Pseudaminobacter*, *Burkholderia*, *Shinella*, *Arthrobacter*, *Bacillus*	root nodules	[33]
*Mesorhizobium*, *Methylobacillus*, *Arthrobacter*, *Bacillus*, *Rhodococcus*, *Ramlibacter*, *Janthinobacterium*, *Kaistobacter*, *Rubrobacter*	root	[33]
*Klebsiella*, *Pantoea*, *Staphylococcus*, *Rhizobium*, *Stenotrophomonas*, *Enterobacter*, *Paenibacillus* sp., *Bacillus* sp., *Pseudomonas* sp.	root	[26]
*Achromobacter xylosoxidans*, *Bacillus cereus*, *Bacillus thuringiensis*, *Bacillus subtilis*	root	[15]
*Paenibacillus* sp.	root	[34]
*Mesorhizobium* sp.	root	[35]
*Priestia megaterium*, *Brucella haematophila*, *Microbacterium paraoxydans*	root	[36]
*Pseudomonas putida*, *Pseudomonas alcaligenes*, *Pseudomonas*	root	[37]
*Mesorhizobium* sp.	root	[38]
*Mesorhizobium huakuii*, *Mesorhizobium amorphae*	root	[39]
*Bacillus cereus*, *Achromobacter xylosoxidans*, *Bacillus thuringiensis*, *Bacillus subtilis*	root	[40]
*Streptomyces* sp.	roots, stems, leaves	[41]
*Bacillus altitudinis*	rhizosphere	[42]
*Bacillus subtilis*, *Bacillus thuringiensis*, *Bacillus megaterium*	rhizosphere	[43]
*Pseudomonas* sp., *Rhizobium* sp.	rhizosphere	[44]
*Serratia marcescens*, *Pseudomonas fluorescens*, *Rahnella aquatilis*, *Bacillus amiloliquefaciens*	rhizosphere	[45]
*Mesorhizobium ciceri*, *Mesorhizobium loti*, *Mesorhizobium mediterraneum*	rhizosphere	[46]
*Pseudomonas* sp., *Bacillus* sp.	rhizosphere	[47]

Until now, many endophytes were isolated via cultivation methods or identified via cultivation-independent methods from different parts of the chickpea (see Table 1). Recent developments in sequencing technologies allowed scientists to explore the complex nature of microbe-microbe and plant-microbe interactions involving the diverse inhabitants of the chickpea. Despite the technological improvements in the microbiology of bio-inoculation, the conditions and mechanisms behind successful plant-microbe establishment are largely unknown.

This review focuses on the current knowledge of bacterial endophytes of chickpeas, their origin, plant growth-promoting features, and their capacity to buffer abiotic and biotic challenges. Moreover, it will discuss the future perspectives and challenges of using endophytes for improving chickpea performance under drought- and salt-affected lands.

## 2. Endosphere colonization of endophytes and endophytic microbes associated with chickpeas

Plant endophytes can colonize the inner tissues of plants either via horizontal transfer or vertical transfer. It is referred to as a horizontal endophyte transfer when microbes originate from environmental sources such as soil, water, air, or other organisms, while it is referred to as a vertical transfer when microbes are inherited via seeds or plants germinating organs [48]. Apart from the vertical and horizontal transfer, endophytes can enter and establish within plant tissues via the stomata, hydathodes, lenticels, and wounds caused by a pathogen or mechanical damage [49]. However, plants can not recognize endophytes as endophytes may penetrate plants using the same strategy as pathogenic microorganisms as shown by Johnson et al. [50] with *Neotyphodium coenophialum*, an endophyte found in tall fescue, which colonizes the intercellular tissues of the plant without triggering a defense response [50]. Therefore, plant host species or genotype selection [51], and the ability of bacteria to adapt to the inner environment of the host plant [52], are important parameters for colonization success. Furthermore, regulation of receptor-like kinases necessary for nod factor recognition appears to be critical to allow rhizobial infection, and this process may be associated with the recruitment of these receptors to membrane microdomains [53]. Soil, geolocation [30],[54], and plant genotype [40],[55] are important factors in determining root endophyte communities. For example, a study by De Meyer and colleagues [56] showed different environmental parameters as drivers of the endophytes in legume nodules.

Endophytic bacteria have been found in various parts of the chickpea plant, including roots, stems, leaves, and seeds. These bacteria establish a symbiotic relationship with the chickpea, where both the plant and the endophyte benefit from the association [57],[58]. One of the most well-known groups of endophytes associated with chickpeas is the genus *Rhizobium*. *Rhizobium* and other symbiotic nitrogen fixers genera, globally indicated as “rhizobia”, form a mutualistic relationship with leguminous plants, such as chickpeas, by infecting the root nodules and fixing atmospheric nitrogen into a form that the plant can use for growth. This nitrogen fixation process is crucial for enhancing the chickpea's productivity and nitrogen nutrition. Apart from rhizobia, other bacteria, such as *Acinetobacter*, *Bacillus*, *Pseudomonas*, *Burkholderia*, *Enterobacter*, *Microbacterium*, *Paenibacillus*, *Pantoea*, *Arthrobacter*, *Kosakonia*, *Klebsiella*, *Stenotrophomonas*, and *Streptomyces*, have been reported as endophytes in chickpeas [26],[29],[30],[33],[36]. Most of these genera are also reported as endophytes of other plant species such as wheat, barley, rice, maize, and the common bean [59]–[62]. *Mesorhizobium* is the most dominant genus in root nodules of chickpeas, also identified in the seed and root [29], indicating an interspecific interaction between this microbe and the chickpea plant. There are very few reports about the fungal endophytic microbiome of chickpeas where most of them studied AM and non-AM fungal endophytes [63].

## 3. Plant growth stimulation and abiotic stress tolerance

The role of PGPR in mitigating abiotic stress via multiple mechanisms is well-known. For example, the synergistic application of PGPRs—*Bacillus subtilis*, *Bacillus thuringiensis*, and *Bacillus megaterium*—and plant growth regulators—salicylic acid and putrescine—effectively enhances physiological parameters such as chlorophyll, protein, and sugar contents, while also aiding in osmoregulation, ameliorating oxidative stresses, and inducing new proteins, ultimately promoting plant growth and tolerance to environmental stressors [64]. Furthermore, exopolysaccharides synthesis protects the plant from both desiccation and different pathogens/predators via the formation of a rhizosheath around the roots [65],[66]. Besides, interaction with plants leads to the exudation of various biochemical compounds that enhance nutrient uptake and increase plant resistance to pathogens. However, little is known about endophytes associated with chickpeas in terms of drought and salt tolerance. Many culturable endophytic bacteria of chickpeas have shown growth-promoting effects on host plants [67] by producing phytohormones such as auxin, gibberellin, salicylic acid, jasmonic acid, and cytokinin [68]. Auxin stimulates plant cell expansion and proliferation as well as, indirectly, root and shoot growth [69]. Cytokinin and auxin are key regulators of nodule initiation in the root cortex. Salicylic acid also plays an important role in improving resistance to abiotic and biotic stress in chickpeas via regulating the antioxidative enzyme activities such as polyphenol oxidase and peroxidase [68]. The metabolic profile of drought stress-tolerant varieties showed a significant increase in catalase, ascorbate peroxidase, and peroxidase activities as compared to sensitive varieties under stress conditions [65],[70]. This might indicate that endophyte-produced molecules might induce antioxidative enzyme activities under drought stress. However, it is necessary to assess how consistently different endophytes exhibit beneficial traits across various chickpea cultivars or environmental conditions.

In plant cells under drought stress, reactive oxygen species (ROS) accumulate and cause oxidative damage and cell death [71]. Antioxidant enzyme activities are known to increase in conditions of drought, salt, low temperature, and heavy metal exposure [72] and lessen oxidative damage, thus helping plants build an antioxidant defense system. Further, endophytes play a role in lowering plant ethylene levels, which is a typical plant response to various environmental challenges [73] by producing the enzyme 1-aminocyclopropane-1-carboxylic acid (ACC) deaminase encoded by the *acdS* gene. Inoculation with the ACC-deaminase producers *Arthrobacter nitroguajacolicus* and *Harmannibacter diazothrophicus* increased resistance against salt stress in wheat [74] and barley [75], respectively.

Endophytes are often used in the co-inoculation of multiple endophytes or with AMF as facilitators [29] under water-limited conditions. The mechanisms of how co-inoculation works, however, have not been explained so far. For instance, the co-inoculation of chickpeas with root endophyte *Mesorhizobium* sp. and AMF has increased the protein content of chickpea grains under water-deficit conditions [76].

## 4. Use of endophytes as biocontrol agents

The chickpea is susceptible to various diseases that can affect its productivity and quality. For example, *Fusarium* wilt, caused by the soilborne fungus *Fusarium oxysporum* f. sp. *ciceris* is a significant disease of chickpeas worldwide. It can cause severe yield losses in susceptible varieties. The pathogen infects the root system of the plant and spreads upward through the vascular tissue, leading to wilting, yellowing, and eventually plant death [47]. Root rot is a common disease of chickpeas caused by various soilborne pathogens, including fungi like *Rhizoctonia solani*, *Fusarium* spp., and *Pythium* spp. The disease manifests as root decay, discoloration, and reduced root mass. Infected plants may exhibit wilting, stunting, and overall poor vigor, ultimately resulting in yield losses [77]. Further soil-borne pathogens such as *Phytophthora medicaginis*, *Pythium irregulare*, and *Botrytis cinerea* can cause severe diseases and significantly reduce yield in chickpeas.

Endophytes can be used as biocontrol agents to suppress pathogens: for example, *Streptomyces*, *Bacillus*, and *Pseudomonas* strains are well-known to be effective biocontrol candidates [35],[78]. Apart from bacterial strains, fungal microbes are also effectively used against root rot diseases such as *Penicillium*, *Cladosporium*, and *Fusarium* [79]. Fatima et al. [80] found that 17 bacterial isolates out of 50 demonstrated antagonistic activity against *Fusarium oxysporum*. Among them, *Serratia* sp. and *Enterobacter* sp. significantly suppressed the chickpea wilt and improved root system and plant biomass. Similar results were observed by Mukherjee et al. [18], who showed that the endophytes *Bacillus subtilis* and *Enterobacter hormaechei* increased the tolerance of chickpea plants to the pathogen *Fusarium oxysporum* f. sp. *ciceris*. In addition, out of the 255 bacterial endophytes that were isolated from seven different crop plants (chickpea, tomato, wheat, berseem, mustard, potato, and green pea), three isolates were found to have strong inhibition (>50%) against three fungal pathogens: *R. solani*, *Sclerotium rolfsii*, and *F. oxysporum* f. sp. *ciceri*. These three endophytic isolates were then characterized using morphological, biochemical, and molecular methods and were identified as different strains of *Bacillus subtilis*. The beneficial effects of these bacteria on chickpea plants were elucidated by their capacity to reduce the prevalence of disease, stimulate growth parameters, and significantly enhance root characteristics. They also exhibited a high level of colonization of the roots of endophyte-inoculated plants, a reduction in the production of superoxide, an enhancement of plant defense enzymes, and an induction of the expression of pathogenesis-related genes through seed priming [81]. Another study, conducted by Gorai et al. [82], demonstrated the potential of endophytic *Bacillus siamensis* CNE6 isolated from the nodule of chickpeas to control black root rot disease of *C. arietinum* L. The researchers found that this endophytic bacterium produced a secondary metabolite called (2E)-6-methoxy-2-[(4-methoxyphenyl) methylidene]-2,3-dihydro-1-benzofuran-3-one, which is effective in inhibiting the activity of lanosterol 14-alpha demethylase, an enzyme involved in both ergosterol biosynthesis and beta-tubulin assembling in the fungal pathogen that causes the disease. In addition, endophytic *Bacillus siamensis* CNE6 also upregulated the expression of four defense genes (CHI1, PAMP, PR2B, and TF1082) in *C. arietinum* upon pathogenic challenge in in vivo experiments.

## 5. Functional traits associated with plant health and fitness

In the plant rooting zones, PGPR synthesize a variety of phytochemicals, such as siderophores, cell wall-degrading enzymes, antimicrobial metabolites, hydrogen cyanide, and phytohormones [16]. PGPR also play a vital role in inducing defense mechanisms against pathogens [73]. These rhizobacteria can activate specific pathways and signaling molecules in the plants, which result in the production of defense enzymes and phytochemicals. One particular pathway involved in the induction of defense enzymes is the jasmonic acid pathway. Upon perception of conserved elicitor molecules from the rhizobacteria, the plant activates the jasmonic acid signaling pathway. This pathway is initiated by the activation of receptors, such as FLS2 (Flagellin Sensing 2) or EFR (elongation factor Tu receptor), which recognize microbial-associated molecular patterns (MAMPs) released by the rhizobacteria [73]. The subsequent activation of these receptors initiates a signaling cascade, which ultimately leads to the production of jasmonic acid, a phytohormone that is involved in the regulation of plant defense responses. Jasmonic acid then acts as a signaling molecule, activating the expression of genes involved in the synthesis of defense enzymes [73]. These defense enzymes include chitinases, glucanases, peroxidases, and proteases, which play a vital role in breaking down and destroying pathogens or their components [73]. Several bacterial strains that showed the biological control ability of chickpea pathogens demonstrated the production of hydrogen cyanide, and cell wall-degrading enzymes such as glucanase, chitinase, protease, and siderophore [18]. For example, *Enterobacter hormaechei* produces Indole-3-acetic acid, and *Bacillus subtilis* solubilizes phosphate and potassium and inhibits the *Fusarium* pathogen. Several important defense enzymes, such as phenylalanine ammonia-lyase, peroxidase, polyphenol peroxidase, and -1,3 glucanase, were expressed in chickpea plants after being inoculated with *Serratia* or *Enterobacter* strains. These enzymes may have helped the plants resist the pathogen invasion [80]. In a study conducted by Sreevidya et al. [83], about 89 actinomycetes were screened for their ability to antagonize fungal pathogens of chickpeas using dual culture and metabolite production assays. The four most promising actinomycetes were then evaluated for their physiological and plant growth-promoting properties under both in vitro and in vivo conditions. They were also found to produce siderophores, cellulase, lipase, protease, chitinase, hydrocyanic acid, indole acetic acid, and β-1,3-glucanase. Expression profiles for indole acetic acid, siderophore, and β-1,3-glucanase genes were upregulated for all three traits and in all four isolates.

**Figure 1.**
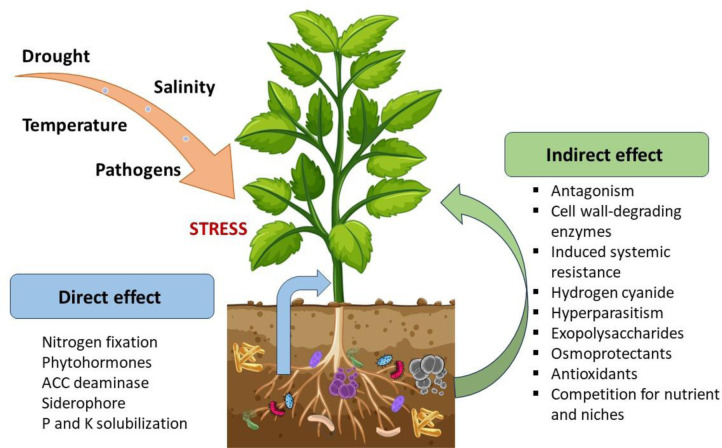
Plant beneficial traits of endophytic bacteria in promoting plant growth and the biological control of plant pathogens.

Furthermore, the leaves of the chickpea inoculated with the selected endophytes showed induction of antioxidant enzymes such as superoxide dismutase, catalase, ascorbate peroxidase, guaiacol peroxidase, glutathione reductase, phenylalanine ammonia-lyase, polyphenol oxidase, and phenolics, compared to the uninoculated control [41]. Similar results were demonstrated by Abd Allah and colleagues [84], who observed enhanced plant biomass, reduced levels of reactive oxygen species (ROS), and lipid peroxidation in plants under salt stress. *Bacillus subtilis* (BERA 71) increased the superoxide dismutase, peroxidase, catalase, and glutathione reductase activities, as well as the levels of non-enzymatic antioxidants such as ascorbic acid and glutathione. It has been indicated that the suppression of ROS generation of lipid peroxidation, and the accumulation of proline in inoculated plants with plant-beneficial bacteria enhance membrane stability under abiotic stress conditions. Table 2 and Figure 1 demonstrate plant beneficial traits of endophytic bacteria.

**Table 2.** Plant beneficial traits of endophytic bacteria associated with chickpeas.

Bacterial endophytes	Plant benefits	Mechanism	Reference
*Bacillus subtilis*	Plant growth promotion	Production of siderophore, IAA, accumulation of superoxide, enhanced the plant defense enzymes, induced the expression of pathogenesis-related genes	[81]
*Achromobacter xylosoxidans*, *Bacillus cereus*, *Bacillus thuringiensis*, *Bacillus subtilis*	Plant growth promotion and stress tolerance, yield	Production of siderophore, IAA, HCN, cell wall-degrading enzymes, and compatible osmolytes	[15]
*Enterobacter hormaechei*, *Bacillus subtilis*, *Pseudomonas aeruginosa*	Plant growth promotion	Production of siderophore, IAA, cell wall-degrading enzymes, involved in phosphorus and potassium solubilization	[25]
*E.cloacae*, *E. hormaechei*, *B. subtilis*	Antagonistic activity against *Fusarium oxysporum*, plant growth promotion	Production of siderophore, IAA, ammonium, cell wall-degrading enzymes	[18]
*Bacillus* sp. *Mesorhizobium* sp. *Burkholderia* sp.	Plant growth promotion	Production of siderophore and involved in phosphorus solubilization	[29]
*Mesorhizobium* sp.	Plant growth promotion	IAA production, involved in phosphorus solubilization	[32]
*Actinobacteria* sp., *Enterobacter* sp., *Pseudomonas* sp., *Pantoea* sp., *Rhizobium* sp., *Stenotrophomonas* sp.	Plant growth promotion and antagonistic activity against fungal pathogens	Production of IAA and ammonia	[26]
*Mesorhizobium ciceri*, *Streptomyces* sp.	Plant growth, yield, biological control of *Botrytis* grey mold	Production of antioxidant enzymes, superoxide dismutase, catalase, ascorbate peroxidase, guaiacol peroxidase, glutathione reductase, phenylalanine ammonia-lyase, polyphenol oxidase, and phenolics	[41]
*Bacillus siamensis*	Plant growth promotion	Production of siderophore, IAA, induction of antagonistic and ACC-deaminase activities, involved in phosphate solubilization and nitrogen fixation	[82]

## 6. Genes involved in plant-microbe symbiotic-mutualistic interactions

The chickpea is one of the oldest plants that was domesticated around 11,000 years ago along with wheat, barley, the common bean, peas, and lentils [85]. The strains of *Rhizobium* sp. isolated from wild chickpea (*Cicer anatolicum*) showed good performance and effective symbiosis when chickpea plants were inoculated and grown under low and normal temperature conditions. Genes involved in the rhizobial symbiosis of chickpeas seem to be very conserved maybe due to its long cultivation history [32]. Hence, compared to other crops with a relatively recent history of cultivation, the chickpea genome gathers genetic information about forming stable positive connections with their associated microorganisms.

The bacterial genes involved in symbiosis can be split into two groups: 1) nodulation genes (*nod*) such as *NodC*-chitin synthase, *NodB*-N-deacetylase, and *NodA*-acyl transferase which are involved in nodulation, and 2) nitrogen fixation genes (*nif*), which are involved in atmospheric nitrogen fixation. The Nod factor assembly process begins with the *NodC* gene, which is also a host factor and necessary for nodulation in all rhizobia. Despite *nod* genes being specific to rhizobia, *nif* genes are widespread in bacteria. Chickpea is considered a restrictive host which recognizes only a few Nod factors that are highly conserved among rhizobia species. *nodC* or *nifH* genes are therefore used in many studies as an indication of rhizobia symbiosis in chickpea [32]. The absence of these gene sequences can be used to identify nodules inhabiting non-rhizobial endophytes. De Meyer et al. [56] found in the nodules of the majority of the legume species relatively more rhizobial than non-rhizobial endophytes. They also observed the co-occurrence of certain rhizobia with particular non-rhizobial endophytes, which suggests inter-specific microbe-microbe interactions [56]. Apart from nod genes, the ClpB chaperone protein is also important in bacterial stress response, and recent research suggests it also plays a role in chickpea-rhizobia symbiosis [86]. The *Mesorhizobium mediterraneum* UPM-Ca36T strain was genetically enhanced with an additional copy of the *clpB* gene, and this modified strain showed increased tolerance to heat and acid stress, and showed improved symbiotic performance with chickpeas compared to the unmodified strain [87]. This suggests that the modification of these genes might be an effective method to create rhizobial strains with both increased stress resilience and symbiotic capabilities, potentially improving their use as crop inoculants under environmental stress.

There are many culturable rhizobial and non-rhizobial endophytes isolated, however often they fail to form nodules in the legume from which they were isolated. Hence, they might not have directly contributed to the development of the nodules, but they may have indirectly initiated the nodulation by producing signaling molecules. Intracellular bacteroids which thrive inside the nodule cells can be taken as an example. Although rhizobia initiates nitrogen-fixing nodules on its leguminous host plant, bacteroids are located inside the nodule cells, and convert atmospheric nitrogen into ammonia [88]. The physiological state of intracellular bacteria differs from that of their free-living counterparts, and they must adjust to the environment in which they live, including oxidative stress, a microoxic environment, a low pH environment, and certain carbon and nitrogenous substances [88]. The successful colonization of such harsh habitats by bacteria is required to adapt to environmental conditions. One of the survival mechanisms of bacteria is the stringent response. This conserved regulatory mechanism is controlled by the alarmone (p) ppGpp, which regulates physiological adaptations to nutrient starvation and other stresses [89]. The synthesis of these molecules is regulated by the *rsh* gene (encodes SelA, SpoT proteins). A stringent response is also required for nodule formation in legumes such as *Phaseolus vulgaris* and *Medicago sativa* [90],[91]. For example, ppGpp was shown to accumulate in symbiotic bacteria, including *Sinorhizobium meliloti*, as a result of amino acid starvation [91]. When the synthesis of (p) ppGpp was interrupted by deleting the *rsh* gene in *Rhizobium etli*, the nitrogen fixation activity in nodules is significantly reduced as well as resulting in changes in *R. etli* bacteroids' physiology [90]. These studies show the microbe-microbe and microbe-host interspecific interactions in response to environmental stress conditions.

## 7. Challenges in harnessing endophytes for drought tolerance in chickpeas

To improve drought tolerance in crops, such as chickpeas, by exploiting endophytic microbes, breeders and farmers must face significant challenges, such as:

- Identifying and characterizing effective endophytic strains with consistent and reproducible drought-resistance-promoting abilities.
- Understanding the intricate molecular communication between hosts and endophytes under drought stress conditions. This understanding will enable breeders to fully harness the potential of endophytes in enhancing drought tolerance in chickpeas.
- Translating laboratory findings into field applications, which requires addressing practical issues such as scalability, stability, and compatibility, among others.
- The use of accurate and relevant phenotyping techniques to both select drought-resilient genotypes and understand the genetic landscape underlying the adaptive response of chickpeas to drought.

By integrating the knowledge gained from phenotyping with the understanding of endophyte-mediated mechanisms, breeders can develop drought-tolerant chickpea cultivars that can withstand water-limited conditions and contribute to increased food production.

## 8. Conclusion and future perspectives

Using seed or root endophytes as bio-inoculants for chickpeas might be more efficient than using soil or rhizosphere bacteria, due to fast recognition by the host plant as a result of co-evolution [60]. The long history of chickpea cultivation might lead to establishing more mature interactions between its endophytic microbes. According to the holobiont theory, plants and their associated microbiome are considered as a single biological unit exposed to evolutionary processes. This suggests that plants have evolved strategies for differentiating their evolutionary partners from other microorganisms. Therefore, the use of endophytes as inoculants for improving chickpea performance in arid areas has potential. Especially, searching for potential candidates among those who have a strong connection with chickpea plants can be more effective. On the other hand, non-rhizobial endophytic bacteria in legumes should also be studied for successful rhizobia inoculation as those endophytes might have indirect stimulations and play important roles in the symbiotic interactions. As was shown in several studies, co-inoculation of rhizobia with other PGPRs such as *Pseudomonas*, *Bacillus*, *Azotobacter*, *Erwinia*, *Serratia*, etc. can be more efficient in decreasing abiotic stresses in legumes [92]. When exposed to abiotic stressors, these bacteria might also be responsible for up- or downregulating some genes linked to the production of plant metabolites, which might lead to crop plants developing resistance against drought. Endophytic bacteria are potential candidates as biotechnological tools that could (at least in part) displace conventional agrochemicals. Based on previous reports, endophytic bacteria such as *Bacillus*, *Mesorhizobium*, and *Burkholderia* are frequently found as more abundant microbes regardless of geographical location, which suggests that they may be vertically transferred from seeds to plants over generations and establish a solid relationship with the host plant. Comprehending and understanding the plant microbiota in connection to plant genotype and environmental factors may lead us to develop more effective inoculation techniques of chickpeas in each specific agronomical situation.

In conclusion, the symbiotic association between chickpea plants and their endophytes presents a compelling strategy for enhancing drought resilience, a critical factor in sustaining chickpea production under varying climatic conditions. By elucidating the mechanisms through which endophytes confer stress tolerance and improve plant health, researchers can pave the way for innovative agricultural practices that leverage these microbial interactions. Further exploration and exploitation of chickpea endophytes hold promise not only in fortifying this vital crop against environmental stressors but also in contributing to the broader goals of sustainable agriculture and food security.

## Use of AI tools declaration

The authors declare they have not used Artificial Intelligence (AI) tools in the creation of this article.
